# High Levels of Transmitted HIV Drug Resistance in a Study in Papua New Guinea

**DOI:** 10.1371/journal.pone.0170265

**Published:** 2017-02-01

**Authors:** Evelyn Lavu, Ellan Kave, Euodia Mosoro, Jessica Markby, Eman Aleksic, Janet Gare, Imogen A. Elsum, Gideon Nano, Petronia Kaima, Nick Dala, Anup Gurung, Silvia Bertagnolio, Suzanne M. Crowe, Mark Myatt, Anna C. Hearps, Michael R. Jordan

**Affiliations:** 1 Central Public Health Laboratory, Port Moresby, Papua New Guinea; 2 Centre for Biomedical Research, Burnet Institute, Melbourne, Victoria, Australia; 3 Institute for Medical Research, Goroka, Papua New Guinea; 4 National Department of Health, Port Moresby, Papua New Guinea; 5 Mount Hagen Hospital, Mount Hagen, Papua New Guinea; 6 World Health Organization, Port Moresby, Papua New Guinea; 7 HIV Department, World Health Organization, Geneva, Switzerland; 8 Brixton Health, Llawryglyn, Powys, Wales, United Kingdom; 9 Division of Geographic Medicine and Infection Disease, Tufts Medical Center, Boston, Massachusetts, United States of America; 10 Tufts University School of Medicine, Boston, Massachusetts, United States of America; Universidad Autonoma de Madrid Centro de Biologia Molecular Severo Ochoa, SPAIN

## Abstract

**Introduction:**

Papua New Guinea is a Pacific Island nation of 7.3 million people with an estimated HIV prevalence of 0.8%. ART initiation and monitoring are guided by clinical staging and CD4 cell counts, when available. Little is known about levels of transmitted HIV drug resistance in recently infected individuals in Papua New Guinea.

**Methods:**

Surveillance of transmitted HIV drug resistance in a total of 123 individuals recently infected with HIV and aged less than 30 years was implemented in Port Moresby (n = 62) and Mount Hagen (n = 61) during the period May 2013-April 2014. HIV drug resistance testing was performed using dried blood spots. Transmitted HIV drug resistance was defined by the presence of one or more drug resistance mutations as defined by the World Health Organization surveillance drug resistance mutations list.

**Results:**

The prevalence of non-nucleoside reverse transcriptase inhibitor transmitted HIV drug resistance was 16.1% (95% CI 8.8%-27.4%) and 8.2% (95% CI 3.2%-18.2%) in Port Moresby and Mount Hagen, respectively. The prevalence of nucleoside reverse transcriptase inhibitor transmitted HIV drug resistance was 3.2% (95% CI 0.2%-11.7%) and 3.3% (95% CI 0.2%-11.8%) in Port Moresby and Mount Hagen, respectively. No protease inhibitor transmitted HIV drug resistance was observed.

**Conclusions:**

The level of non-nucleoside reverse transcriptase inhibitor drug resistance in antiretroviral drug naïve individuals recently infected with HIV in Port Moresby is amongst the highest reported globally. This alarming level of transmitted HIV drug resistance in a young sexually active population threatens to limit the on-going effective use of NNRTIs as a component of first-line ART in Papua New Guinea. To support the choice of nationally recommended first-line antiretroviral therapy, representative surveillance of HIV drug resistance among antiretroviral therapy initiators in Papua New Guinea should be urgently implemented.

## Introduction

As of 2016, 17 million people were receiving antiretroviral therapy (ART) in low- and middle-income countries [1]. Papua New Guinea (PNG) has the highest prevalence of HIV among the Pacific Island nations with an estimated national prevalence in 2016 of 0.8% amongst adults aged 15 to 49 years [2]. PNG’s HIV epidemic is characterized as “concentrated” in certain geographical locations and within certain key population groups rather than being generalized within the adult population. Studies indicate a high prevalence of HIV amongst female sex workers, with most recent studies reporting a prevalence of 19% in Port Moresby [3]. The HIV prevalence amongst male sex workers in Port Moresby is also high at 8.8%, and 23.7% of transgender males who sell sex are infected with HIV [3].

ART was introduced in PNG in 2004, and by 2015 treatment coverage was estimated to be 53% (*n* = 21,198) of those in need [2]. ART is provided using a public health model of care. CD4 cell count testing is available, often at point of care, in certain parts of the country, but is still being scaled-up nationally. Likewise, access to viral load monitoring is slowly expanding. HIV drug resistance (HIVDR) genotyping is not performed in country [4] and is not available for clinical decision making. In PNG, currently recommended first-line ART consists of two nucleoside reverse transcriptase inhibitors (NRTI) (zidovudine + lamivudine or tenofovir + lamivudine) in combination with a non-nucleoside reverse transcriptase inhibitor (NNRTI), either efavirenz or nevirapine. Second-line ART consists of zidovudine +lamivudine or tenofovir + lamivudine (using the NRTI not prescribed in first-line ART), in combination with lopinavir/ritonavir, a boosted protease inhibitor (PI) [2].

Some HIVDR is expected to emerge in populations receiving ART even with optimal adherence to therapy. This drug resistant virus can be transmitted to previously uninfected individuals or rarely, in the case of HIV superinfection, to previously infected individuals. Population-level monitoring of transmitted HIV drug resistance (TDR) can inform the selection of antiretroviral (ARV) drugs for inclusion in national ART regimens. The most suitable population to survey for TDR is recently infected individuals who are unexposed to ARV drugs because, over time and at variable rates, resistance-associated mutations revert to wild-type or fall below the level of detection of standard genotyping assays [5].

In a pooled analysis of TDR surveys published in the World Health Organization’s (WHO) 2012 global HIVDR report, the estimated prevalence of TDR to NNRTI increased between 2004 and 2010 [1]. This increase was particularly apparent in the areas surveyed in Africa, where the prevalence of NNRTI resistance reached 3.4% (95% CI 1.8%-5.2%) [6]. More recently, higher levels of drug resistant virus have been observed amongst people naïve to ARV drugs in Angola (16.3%), Botswana (9.7%), Cuba (14.8%), Mexico (12.0%), and South Africa (14.2%) [7,8,9,10,11].

To address the threat of HIVDR to the success of ART scale-up, the WHO developed a global surveillance and monitoring strategy in 2004, subsequently updating it in 2015 [12]. Between 2004–2013, WHO recommended surveillance of TDR among HIV-infected individuals likely to have been recently infected (within three years of diagnosis) and unlikely to have been exposed to ARV drugs [13]. The WHO-recommended approach targeted geographic regions within a country where TDR was likely to be observed first. Specifically, areas with high levels of ART coverage and longer availability of ART were preferentially surveyed, with the goal of providing an alert to policy makers and stakeholders that transmission of drug-resistant virus was occurring [13].

In 2013–2014, following WHO guidance, PNG implemented surveillance of TDR in two geographic regions with high HIV prevalence: Port Moresby, National Capital District, and Mount Hagen, Western Highlands Province. Port Moresby and Mount Hagen report a HIV prevalence of 1.0% and 1.3%, respectively [2]. As the nation’s capital city, Port Moresby is an urban area and is the country’s major economic and transit hub; Mount Hagen is a regional center located in the highlands. The specific goal of these surveys was to estimate the level of TDR to the NRTI, NNRTI and PI drug classes amongst voluntary counseling and testing (VCT) or sexually transmitted infection (STI) clinic attendees likely to have been recently infected with HIV and unlikely to have been exposed to ARV drugs in these two regions.

## Materials and Methods

### Survey inclusion criteria and survey sites

Generic WHO guidance recommends the genotyping of specimens from ARV naïve men or women, aged–less than 25 years, and if female, primiparous or never pregnant [13]. These epidemiological criteria are used to maximize the likelihood that genotyping is performed on specimens from individuals likely to have been recently infected and to be ARV naïve. However, due to the older age at time of HIV infection in males and females in PNG (median age 33 years and 27 years, respectively) [14], when compared to generic WHO TDR survey guidance, the survey age criterion was modified in consultation with WHO and country-level experts to ensure recruitment of a sufficient number of members of the eligible population within a meaningful timeframe. In these surveys, individuals were eligible to participate if they were aged 16 years (age of majority in PNG) -30 years, ARV drug naïve, and if female, primiparous or never pregnant. In Port Moresby, two STI clinics were chosen: Heduru HIV/STI clinic, Port Moresby General Hospital and Anglicare Clinic, Waigani, Port Moresby. In Mount Hagen, two VCT centers were chosen: Rebiamul Hospital VCT Center and Tininga Clinic based at Mount Hagen Hospital. As per PNG’s national guidelines, HIV infection was diagnosed by a reactive test on two rapid screening tests (Alere Determine^™^ HIV-1/2 and HIV1/2 STAT-PAK^®^). Where equivocal results were obtained, HIV was confirmed by Serodia^®^-HIV 1/2 and ImmunoComb HIV1&2 assays.

### Ethical review

These TDR surveys were reviewed and approved by the National AIDS Council Research Advisory Board in PNG (No. RES12-018). Ethical approval to perform HIVDR testing in Melbourne, Australia on specimens from individuals of any age collected for these TDR surveys was approved by The Alfred Hospital Ethics Committee (No. 35/05). Participants signed written informed consent to participate in the survey.

### Laboratory methods

After providing informed consent, 10 ml of whole blood was collected in EDTA-containing tubes by venepuncture. Dried Blood Spots (DBS) were made by spotting whole blood onto Whatman^®^ 903 cards. DBS cards were dried and stored at -20°C following WHO laboratory guidance for HIVDR testing [15]. Specimens were sent to the Burnet Institute, Melbourne, Australia (a WHO-designated regional HIVDR testing laboratory) for sequencing. Viral nucleic acids were extracted from two 50μL DBS using BioMérieux NucliSENS^™^ miniMAG manual extraction per manufacturer’s instructions. HIVDR genotyping of the protease and reverse transcriptase regions of the HIV-1 pol gene were obtained using previously described methods [16]. Briefly, the entire protease (PR) gene and amino acids 1–250 of revere transcriptase (RT) were amplified via nested RT-PCR using the primers 2077Fwd (5’-CAGGCTAATTTTTTAGGGAAAATYTGG-3’) and 3402Rev (5’-TCTGTTAGTGCTTTGGYTCC-3’) and the OneStep RT-PCR kit for RT-PCR (Qiagen, Hilden, Germany), and 2142Fwd (5’-CAGACCAGAGCCAACAGCCCCACCAG-3’) and 3346Rev (5’-CCTGSATAAATCTGACTTGCCC) with Platinum Pfx polymerase (Invitrogen, Carlsbad, CA) for the nested PCR (nPCR). nPCR products were purified using the High Pure PCR product purification kit (Roche, Basel, Switzerland) and sequenced with 5pmol of the nPCR primers plus 2997Fwd (5’- CCACAGGGATGGAAAGGATCAC-3’) and 3121Rev (5’- CTATGYTGCCCTATTTCTAAGTCAG-3’) using BigDye version 3.1 sequence chemistry (Applied Biosystems, Foster City, CA, USA). Sequence traces were aligned and consensus sequence constructed using ChromasPro software (Technelysium Pty Ltd, Brisbane, Australia).

Quality assurance (QA) of sequences was performed following WHO-suggested guidance and included the assessment of genetic distance and phylogenetic analysis [17]. Distance measurements and neighbor-joining trees were generated using MEGA 6.0; [18] WHO-suggested HIV reference sequences were used [16]. Distance measurements were used to assess for possible laboratory contamination. HIV-1 subtyping was performed using the Rega Subtyping tool [19,20].

TDR mutations were defined per the 2009 WHO Surveillance Drug Resistance Mutations (SDRM) List [21] with the following caveat: Based on a revised threshold for polymorphisms of 0.2%, M46I and L protease mutations were removed from the SDRM list when performing analysis to be in keeping with revisions introduced at the time of the 2012 WHO HIVDR global report [6]. Exclusion of M46I/L effectively increases the specificity of the analysis although at the potential expense of reduced sensitivity. By reducing the prevalence threshold to differentiate a SDRM from a polymorphism, the proportion of false-positives is reduced and the positive predictive value of the detection of PI resistance is increased.

Point prevalence estimates with 95% confidence intervals (CIs) were calculated for overall and drug-class specific TDR. Differences in prevalence estimates between geographic regions were explored by calculating the prevalence ratio and 95% CI. All calculations were performed using STATA release 13 (StataCorp LP, College Station, TX, USA) [22].

## Results

### Participant demographic characteristics

Overall, 58.5% of survey participants were female (none of whom were pregnant at the time of recruitment) and the median age (IQR) was 25.0 years (21.0–28.0). Demographic characteristics by region and gender are presented in Table 1.

**Table 1:** Demographic characteristics of survey participants.

	Port Moresby	Mount Hagen	Combined
**Gender**	31/62 female (50%)	41/61 female (67%)	72/123 female (58.5%)
**All participants**			
**Age median (IQR)**	25.0 (21.0–28.0)	25.0 (21.0–28.0)	25.0 (21.0–28.0)
**Range**	16–30	16–30	16–30
**Females**			
**Age median (IQR)**	22.0 (20.0–26.0)	22.0 (20.0–27.5)	22.0 (20.0–26.0)
**Range**	18–30	16–30	16–30
**Males**			
**Age median (IQR)**	25.0 (23.0–29.0)	27.5 (22.5–29.8)	27.0 (23.0–29.0)
**Range**	16–30	16–30	16–30

### Transmitted HIV drug resistance—Port Moresby, National Capital District

**Fig 1:**
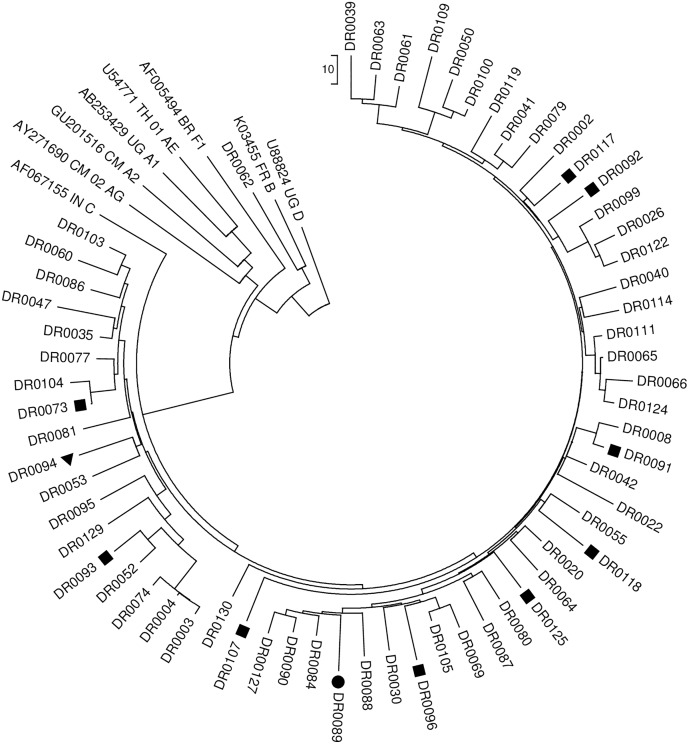
Neighbor-joining tree with WHO Reference Sequences (Port Moresby, National Capital District). Sequences with one of more NNRTI SDRM denoted by squares and sequences with one or more NRTI SDRM denoted by triangles. Sequences with one or more NNRTI and one or more NRTI SDRM are denoted by circles. Subtype reference sequences are included.

Forty-six patients were recruited from Heduru STI clinic during the period 21 May 2013 to 23 April 2014 and 24 patients were recruited from Anglicare Clinic during the period 11 May 2013 to 24 April 2014. Of 70 specimens sent for HIVDR genotyping, 62 (89%) were successfully amplified and passed WHO QA. No sequence was excluded based on WHO-recommended QA criteria. Of the 62 successfully amplified specimens, 10 had one or more NNRTI SDRMs, two had one or more NRTI SDRM, and none had any PI SDRM (see Table 2). Phylogenetic analysis of all sequences was performed and is shown in Fig 1. No clustering of HIV sequences was observed.

**Table 2:** Summary of observed transmitted HIV drug resistance mutations (Port Moresby, National Capital District).

Identification Number	NRTI SDRM	NNRTI SDRM	Gender	Age
DR0073		K103N	M	29
DR0089	M41L,T215Y	K103N	F	21
DR0091		G190A	F	22
DR0092		K103N, V106MV	F	25
DR0093		Y181IV	F	20
DR0094	K70E		M	25
DR0096		K103KN	M	27
DR0107		Y181C	M	30
DR0117		Y181CY	F	22
DR0118		K101E, G190A	M	28
DR0125		V106IV, Y181CY, Y188CY	F	18

SDRM, surveillance drug resistance mutation; NRTI, nucleos(t)ide reverse transcriptase inhibitor;

NNRTI, non-nucleoside reverse transcriptase inhibitor; PI, protease inhibitor

The prevalence of NNRTI and NRTI TDR was 16.1% (95% CI 8.8%-27.4%) and 3.2% (95% CI 0.2%-11.7%), respectively. No PI TDR was observed. Specimens were almost exclusively HIV-1 subtype C (n = 61), with one specimen being HIV-1 subtype B.

### Transmitted HIV drug resistance—Mount Hagen, Western Highlands Province

**Fig 2:**
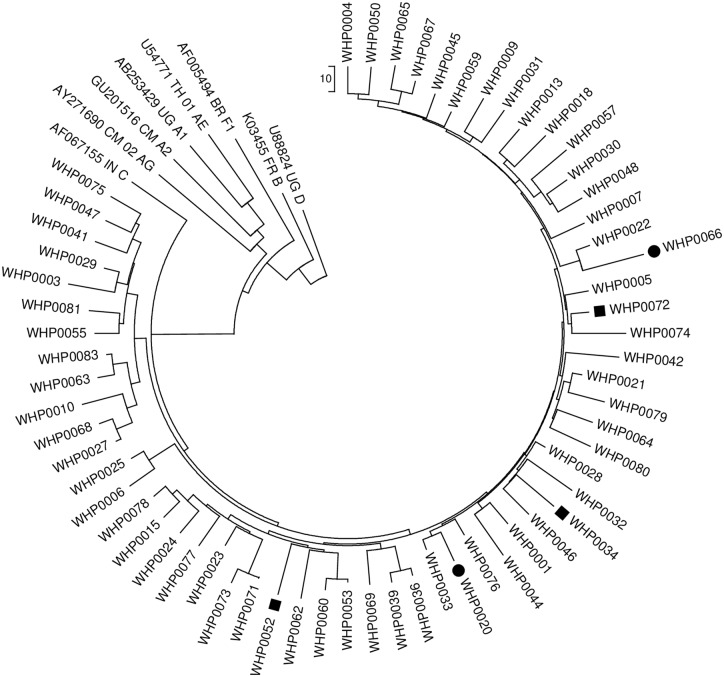
Neighbor-joining tree with WHO Reference Sequences (Mount Hagen, Western Highlands Province Sequences with one of more NNRTI SDRM denoted by squares and sequences with one or more NNRTI and NRTI SDRM are denoted by circles. Subtype reference sequences are included.

Nineteen patients were recruited from Rebiamul VCT clinic during the period 2 August, 2013 to 21 November, 2013 and 51 patients were recruited from Tininga VCT clinic during the period 17 July 19, 2013 to 15 November, 2013. Of 70 DBS shipped for HIVDR genotyping, 63 (90%) were amplified by PCR. Two sequences were subsequently excluded from analysis based on WHO-recommended QA guidance. Specifically, distance measurements suggested that the same individuals may have tested twice (absolute nucleotide distance equal to 1, data not shown). Of the 61 genotyped specimens passing quality assurance, five had one or more NNRTI SDRMs, two had one or more NRTI SDRM, and none had any PI SDRM (see Table 3). Phylogenetic analysis of all sequences was performed and is shown in Fig 2. No significant clustering of HIV sequences was observed.

**Table 3:** Summary of observed transmitted HIV drug resistance mutations (Mount Hagen, Western Highlands Province).

Identification Number	NRTI SDRM	NNRTI SDRM	Gender	Age
WHP0020	K219KN	Y181C, G190A	M	30
WHP0034		K103N	F	30
WHP0052		G190A	M	21
WHP0066	D67N, K70E, M184V	Y181C, G190A	M	25
WHP0072		Y181CY	F	26

SDRM, surveillance drug resistance mutation; NRTI, nucleos(t)ide reverse transcriptase inhibitor; NNRTI, non-nucleoside reverse transcriptase inhibitor; PI, protease inhibitor

The prevalence of NNRTI and NRTI TDR was 8.2% (95% CI 3.2%-18.2%) and 3.3% (95% CI 0.2%-11.8%), respectively. No PI TDR was observed. All specimens were HIV-1 Subtype C.

## Discussion

The high prevalence of NNRTI TDR in Port Moresby (16.1%) and the moderate NNRTI TDR prevalence in Mount Hagen (8.2%) identified in this study are particularly alarming when compared to a previously published regional average of 2.9% [23]. Moreover, they are amongst the highest levels of TDR reported globally to date. Numerous studies demonstrate a significantly higher rate of virological failure in individuals with TDR if prescribed ART comprises at least one drug showing reduced activity [24,25,26]. Therefore, in a setting like PNG with its limited use of viral load monitoring and the absence of individual HIVDR testing prior to treatment initiation, rising levels of TDR are particularly concerning.

Although a previous 2010–2011 estimate of HIVDR in ART-naive treatment initiators in two Highland Provinces was low (2.1%) [15], this previous study did not target recently infected individuals and the age of participants (median age 30, range: 16–62) was higher than in this present study and thus likely underestimated the extent of transmitted resistance. Furthermore, gaps in ART service delivery which favor the emergence and transmission of drug resistant virus, such as suboptimal retention on ART, suboptimal population-level adherence to ART, and drug stock-outs have been documented in PNG. For example, in 2009 loss to follow-up (or unknown treatment outcomes) 12 months after ART initiation reached 32% at one large center and at another large center only 40% of patients picked-up pills on time (a WHO-recommended surrogate measure of population-level adherence to ART) [27]. Neither center attained the WHO-suggested target for loss to follow-up and on time pill pick-up of <15% and >90%, respectively [27]. Moreover, in 2014–2015 retention on ART at 12 months at a different large clinic in PNG was reported to be only 50%, falling well below the WHO-recommended target of >85% [28]. In another recent study, only 40% of HIV-infected patients receiving ART had better than 95% adherence by pill count in the last month at two major sexual health clinics in the PNG highlands [29]. These documented gaps in service delivery combined with anecdotal reports of clinic-level ARV drug stock outs, which may or may not lead to treatment interruption, suggest that conditions may exist within the ART program which favor excess virological failure amongst those receiving ART, the selection of drug resistant virus, and its subsequent onward transmission to previously uninfected individuals.

These surveys in PNG have important limitations. To enhance feasibility of survey implementation in PNG, the maximum age for participant inclusion was increased from 25 years (as recommended by WHO) to 30 years. The inclusion of older individuals may have had the unintended consequence of including chronically infected individuals resulting in an underestimation of TDR. Likewise, it is plausible that older chronically infected individuals could have had undisclosed prior ARV drug exposure, which could have resulted in selection of drug resistant virus; therefore overestimating levels of TDR. However, a sub-analysis of the point prevalence of HIV drug resistance in participants aged less than or equal to 25 years compared to those aged greater than 25 years did not demonstrate significant differences between these two age groups for NRTI or NNRTI resistance at either recruitment site (0>0.05 for all). Additionally, WHO recommends the use of CD4 cell count < 500 cells/mm^3^, when feasible, as a survey exclusion criterion. However, during the survey period, CD4 cell count testing was being introduced in country, and as CD4 cell counts would not have been available for all survey participants, this criterion was not considered.

Investigations at enrolment sites conducted during analysis confirmed that inclusion and exclusion criteria had been rigorously applied. However, the observation of sequences with multi class resistance (one sequence from Port Moresby and two sequences from Mount Hagen) does raise the possibility that some sequences may have been obtained from individuals with previous exposure to ARV drugs. Evidence of two- and three-class TDR has been estimated to occur rarely (0.6%-1.3% and 0.03%-0.4%, respectively) amongst individuals recently infected with HIV and naive to ARV drugs from sub-Saharan Africa, South East Asia, Latin America/Caribbean and upper-income regions [22]. Despite the limitations of these surveys, the populations assessed would, per national policy, initiate standard NNRTI-based ART regardless of prior ARV drug exposure or duration of infection, and thus may at increased risk for treatment failure. Finally, successful genotypic amplification from DBS was 89%; however, it is unlikely that non-amplification was due to an individual’s TDR status, and thus we do not expect a bias.

Results from these two surveys of TDR are derived from a high risk population (STI or VCT clinic attendees who were 30 years old or younger); thus, generalizability to the entire population of recently infected individuals in PNG including men and women over the age of 30 years is uncertain. However, results are relevant to the specific sub-population assessed at the time of the survey and suggest that in this young sexually active population, prevention efforts must be redoubled to decrease HIV incidence and to avert substantially higher levels of NNRTI TDR, which may significantly jeopardize population-level effectiveness of currently available first-line ART in PNG.

The 2016 WHO consolidated guidelines on the use of ARV drugs for treating and preventing HIV infection recommend testing and treating all patients, regardless of CD4 cell count, and the use of pre-exposure prophylaxis (PrEP) for those at high risk of infection [30]. The recommended “Treat All” approach combined with scale-up of PrEP will undoubtedly lead to a decrease in HIV incidence and propel the global community toward the elimination of AIDS as a public health threat. Yet paradoxically, despite these prevention and treatment recommendations, an increase in HIVDR amongst those infected may be observed because as the number of new infections decrease, the proportion due to transmission from people with previous exposure to ARV drugs through prevention of mother to child transmission, prior pre- or post-exposure prophylaxis or previous treatment will increase; therefore, the risk of transmitted HIVDR among the few infected may increase [31,32]. Recent analyses show that the vast majority of TDR in low- and middle-income countries arises from individuals failing first-line ART and not from onward transmission of drug resistant virus within transmission networks [22]; therefore, significant efforts should be made to retain individuals in treatment, support adherence to therapy, reinforce safer sexual practices amongst those receiving ART, and scale-up viral load testing results to identify and rapidly switch patients with virological failure onto different regimens.

Despite limitations, these two surveys of TDR in PNG provide a useful alert that transmission of drug resistant virus is occurring and indeed appears to be occurring at undesirably high levels. In response to these surveys, PNG will implement nationally representative surveillance of pre-treatment HIVDR (PDR) in 2016 following WHO’s updated PDR survey guidance [33]. This survey amongst ART initiators is powered to provide an estimate of overall HIVDR in the population of treatment initiators regardless of previous ARV drug exposure (including Prevention of Mother to Child Transmission, pre- and post-exposure prophylaxis, and previous ART for the treatment of HIV infection) and an estimate of NNRTI resistance in the population naive to ARV drugs. When completed, results of this national PDR survey will support selection of PNG’s recommended first-line ART and prophylactic regimens. Additionally, PDR survey results may inform cost effectiveness analysis of pre-therapy drug resistance testing, accelerate the introduction of globally recommended integrase-containing regimens for all or a subset of the population, prompt a change to PI containing regimens for individuals with prior exposure to ARV drugs, alter the frequency of viral load monitoring and/or enhance population-level adherence counseling [34].

In addition to implementation of routine surveillance of PDR and acquired HIV drug resistance, these survey results highlight the need to scale-up routine assessments of programmatic functioning and development of ART program and public health interventions directed to clinics not achieving global standards of performance [27,28]. Based on previous findings [4,27], specific attention should be paid to strengthening ARV drug procurement and supply chain management to prevent stock outs of ARV drugs, maximizing population adherence to ART and minimizing losses to follow-up, thereby maximizing retention on ART and minimizing potential individual- and population-level treatment interruptions.

## Conclusions

These surveys document unexpectedly high levels of NNRTI TDR in Port Moresby and Mount Hagen, PNG. Results are concerning and although applicable only to the population sampled, may signal higher than expected levels of HIVDR amongst all treatment initiators in PNG. To characterize HIVDR at the national level in 2016, PNG is implementing a nationally representative survey of PDR amongst first-line ART initiators for the purpose of supporting national recommendations for first-line and prophylactic regimens in country. Left unchecked, high levels of HIVDR in populations eligible to initiate ART in PNG may reverse hard won gains in morbidity and mortality and undermine the UNAIDS goal of eliminating AIDS as a public health threat by 2030. In addition to national surveillance of HIVDR, emphasis must be placed on achieving high levels of retention on ART and development of greatly enhanced capacity for in-country HIV viral load testing and use of viral load test results to identify and promptly switch patients with virological failure to different regimens, thereby minimizing the emergence and potential transmission of drug resistant HIV.
